# The Low Incidence of Viral Hepatitis Reactivation Among Subjects on Immunotherapy Reduces the Impact of Suboptimal Screening Rate

**DOI:** 10.3389/fmed.2022.916213

**Published:** 2022-07-15

**Authors:** Laia Aceituno, Juan Bañares, Lourdes Ruiz-Ortega, Ana Callejo-Pérez, Eva Muñoz-Couselo, Carolina Ortiz-Velez, Nely Díaz-Mejía, Ana Barreira-Díaz, María José Carreras, Anna Farriols, María Buti, Mar Riveiro-Barciela

**Affiliations:** ^1^Liver Unit, Hospital Vall d'Hebrón, Barcelona, Spain; ^2^Oncology Department, Instituto de Oncología Vall d'Hebron (VHIO), Hospital Universitari Vall d'Hebron, Vall d'Hebron Barcelona Hospital, Barcelona, Spain; ^3^Pharmacy Department, Vall d'Hebron Hospital, Barcelona, Spain; ^4^Centro de Investigaciones Biomédicas de la Red de Enfermedades Hepáticas y Digestivas, Madrid, Spain

**Keywords:** immunotherapy, checkpoint inhibitors, viral hepatitis, screening, hepatitis B, hepatitis C, cancer, oncology

## Abstract

**Background and Aims:**

Immunotherapy with immune checkpoint inhibitors (ICIs) is a pillar of many advanced tumors. However, there is scarce data concerning the rate of viral hepatitis screening in this population or the risk of viral reactivation.

**Methods:**

Retrospective–prospective study that includes all patients who began ICIs between January/2019 and December/2020 in a University Hospital. Data on viral hepatitis screening prior to the beginning of ICIs were collected. In subjects lacking information, serological tests were requested prospectively. Among HBsAg, anti-HBc, or anti-HCV positive subjects, reactivation was prospectively assessed.

**Results:**

During the 2-year period of study, 595 subjects received ICIs (61.2% male, mean age 63 years). The most prevalent cancers found were 35.5% lung cancer, 12.1% melanoma, and 8.2% head and neck; ICIs schemes were mainly anti-PD1 (65.7%), followed by anti-PD-L1 (19.2%), and combined therapy (13.6%). Prior to immunotherapy, anti-HCV screening was performed in 462 (77.6%) subjects, HBsAg in 462 (77.6%), anti-HBc in 335 (56.3%), and the complete screening in 328 (55.1%). The anti-HBc screening was more frequently ordered among patients treated with concomitant systemic therapy (*p* = 0.003), especially in the case of chemotherapy (*p* = 0.015), though HCV screening was more commonly performed in concomitant therapies different from chemotherapy (*p* = 0.001). Serological tests were completed prospectively in those alive, leading to an overall prevalence for anti-HCV of 3.5%, HBsAg at 1.3%, and anti-HBc of 15.2%. HCV-RNA was detected in 2/19 (both patients with hepatocellular carcinoma), HBV-DNA in 4/7 HBsAg positive, and in 1/75 anti-HBc positive subject. Five out of the 7 HBsAg carriers and 1/75 anti-HBc+ subjects (due to concomitant antiretroviral therapy) received antiviral prophylaxis. Neither cases of HBV reactivation nor changes in HCV viral load were observed.

**Discussion:**

HBV and HCV screening prior to immunotherapy is suboptimal. Though the rate of viral hepatitis reactivation seems extremely low, efforts should be made to optimize viral hepatitis screening prior to immunotherapy for the selection of candidates for either antiviral prophylaxis or periodical follow-up.

## Introduction

Immune checkpoint inhibitors (ICIs) have become a breakthrough in the treatment of many advanced cancers. Immunotherapy with ICIs is based on the use of monoclonal antibodies that target checkpoint molecules, promoting the activation of the immune system and inducing the elimination of metastatic cells (1). The most commonly used mechanisms are cytotoxic T-lymphocyte-associated molecule-4 (anti-CTLA-4), programmed cell death receptor-1 (anti-PD-1), programmed cell death ligand-1 (anti-PD-L1), and anti-LAG3. To date, immunotherapy with ICIs has been approved for more than 17 different cancer types and growing (2).

It is well-known that there is a risk of hepatitis B virus (HBV) reactivation associated with chemotherapy, especially in the hematology setting. Furthermore, chronic hepatitis C is more prevalent in subjects with some solid-organ tumors such as hepatocellular carcinoma but also non-Hodgkin's lymphoma (3). Yet, screening of viral hepatitis is not universal among candidates for chemotherapy, a fact that has led to the development of electronic alerts and platforms for the promotion of diagnosis among physicians prescribing chemotherapy (4). These actions have been based on the poorer prognosis in terms of both morbidity and mortality among patients with HBV reactivation (5, 6). Unlike chemotherapy, data on the effect of immunotherapy on viral hepatitis is scarce and information about the awareness of this topic among ICI-prescribing physicians is lacking. In most registry studies of ICIs, individuals with underlying viral hepatitis were excluded or at least needed to be on nucleos(t)ide analog (NAs) in the case of patients with chronic HBV, to be included. Retrospective studies from real-world cohorts have shown that up to 17% of HBV-infected subjects may suffer from reactivation in cases of immunotherapy without antiviral prophylaxis (7). With regard to resolved HBV infection (isolated anti-HBc+ subjects), few cases of reactivation have been reported so far (8, 9), though data on the real incidence of reactivation remains unknown (10). In contrast to HBV, HCV viral load seems to be unaltered or even reduced by the effect of ICIs (11).

The aim of this study was to analyze the rate of testing for hepatitis B and C before starting ICIs as a way to assess the awareness of Oncologists about viral hepatitis in the population on immunotherapy. In addition, we prospectively estimated the prevalence of viral hepatitis in patients undergoing immunotherapy and the potential risk of viral hepatitis reactivation in this setting.

## Patients and Methods

### Study Design

This is a retrospective–prospective study that included all patients who began oncological immunotherapy between January 2019 and December 2020 at Vall d'Hebrón University Hospital (Barcelona, Spain). Through the electronic records of the Pharmacy Department of our hospital, all patients who began Oncological immunotherapy within the period of study were selected. Data on viral hepatitis screening prior to the beginning of ICIs and demographic characteristics were collected retrospectively. In subjects lacking information and alive at the time of the study (April to December 2021), serological tests were requested prospectively.

This study was approved by the Vall d'Hebron Hospital ethics committee and was conducted in compliance with the principles of the Declaration of Helsinki, Good Clinical Practice guidelines, and local regulatory requirements.

### Data Collection

Data collected retrospectively included patients' demographic characteristics: sex, age, tumor localization, type and date of diagnosis, evidence of prior liver disease, or presence of liver metastases. Regarding therapy, the parameters collected were: previous oncologic treatments (chemotherapy, immunotherapy); current treatment, defined as immunotherapy anti-PD-1, anti-PD-L1, anti-CTLA-4, anti-LAG-3; ICIs discontinuation and reason for discontinuation; concomitant systemic drugs (e.g., chemotherapy or anti-angiogenic drugs), concomitant corticoids. Viral hepatitis screening prior to ICIs consisted of antibodies to hepatitis C virus (anti-HCV), hepatitis B surface antigen (HBsAg), hepatitis B core antibody (anti-HBc), and surface antigen antibodies (anti-HBs). *Complete viral hepatitis testing* included anti-HCV, anti-HBc, and HBsAg performance. Screening for the human immunodeficiency virus (HIV) was also recorded.

It was also recorded in anti-HCV, HBsAg, or anti-HBc positive subjects whether HCV-RNA or HBV-DNA was carried out. Information on concomitant NAs during ICIs was also gathered, as was the reason for the prescription (HIV infection; antiviral prophylaxis). The last update of data was in February 2022.

In subjects lacking viral hepatitis screening and alive at the time of the study, a blood test including HBsAg, anti-HCV, and anti-HBc was prospectively requested for those still on immunotherapy. In subjects positive for either anti-HCV or anti-HBc, HCV-RNA and HBV-DNA were carried out every 6 months to rule out viral reactivation.

### Outcomes

The primary aim of the study was to assess the rate of HBV (HBsAg and anti-HBc) and HCV (anti-HCV) screening prior to immunotherapy as a measure of the Oncologists' awareness of the risk of viral hepatitis reactivation among subjects undergoing ICIs. The secondary endpoint was the assessment of viral reactivation associated with immunotherapy among individuals with viral hepatitis infection: resolved hepatitis C (anti-HCV+/undetectable RNA), active hepatitis C (anti-HCV+/detectable RNA), chronic HBV infection (HBsAg+), past HBV (anti-HBc+). HBV reactivation was defined by the reappearance or rise in HBV-DNA above baseline in patients with chronic hepatitis B, or the appearance of HBV-DNA in the blood or reverse seroconversion to positive HBsAg in those with past HBV infection (isolated anti-HBc+), regardless of the presence of ALT increase (12–14). HCV reactivation was defined as a 2-log increase in HCV-RNA levels compared to baseline. Screening for HIV prior to the beginning of ICIs was also recorded to compare the degree of awareness between viral hepatitis and HIV infection.

### Methods

Serological markers for HBV (HBsAg, anti-HBc, and anti-HBs), HCV, and HIV were analyzed by commercially available electrochemiluminescence immunoassays (COBAS 8,000, Roche Diagnostics, Rotkreuz, Switzerland). Serum viral loads were quantified by an automated real-time PCR COBAS 6,800 (Roche Diagnostics, Mannheim, Germany): HBV-DNA (COBAS HBV test- lower limit of quantification of 20 IU/mL and lower limit of detection-LLD of 10 IU/mL) and HCV-RNA (COBAS HCV test; LLD of 15 IU/mL).

### Statistical Analysis

Normally distributed quantitative variables were expressed as mean and standard deviation (SD) and non-normally distributed as the median and interquartile range (IQR). Categorical variables were expressed as frequencies and percentages and compared using the chi-square or Fisher exact test, as appropriate. The results were considered statistically significant when the *p*-value was below 0.05. All statistical analyses were performed using IBM SPSS, version 26.0 (SPSS Inc, Armonk, NY, USA).

## Results

### Baseline Characteristics of Patients

**Figure 1:**
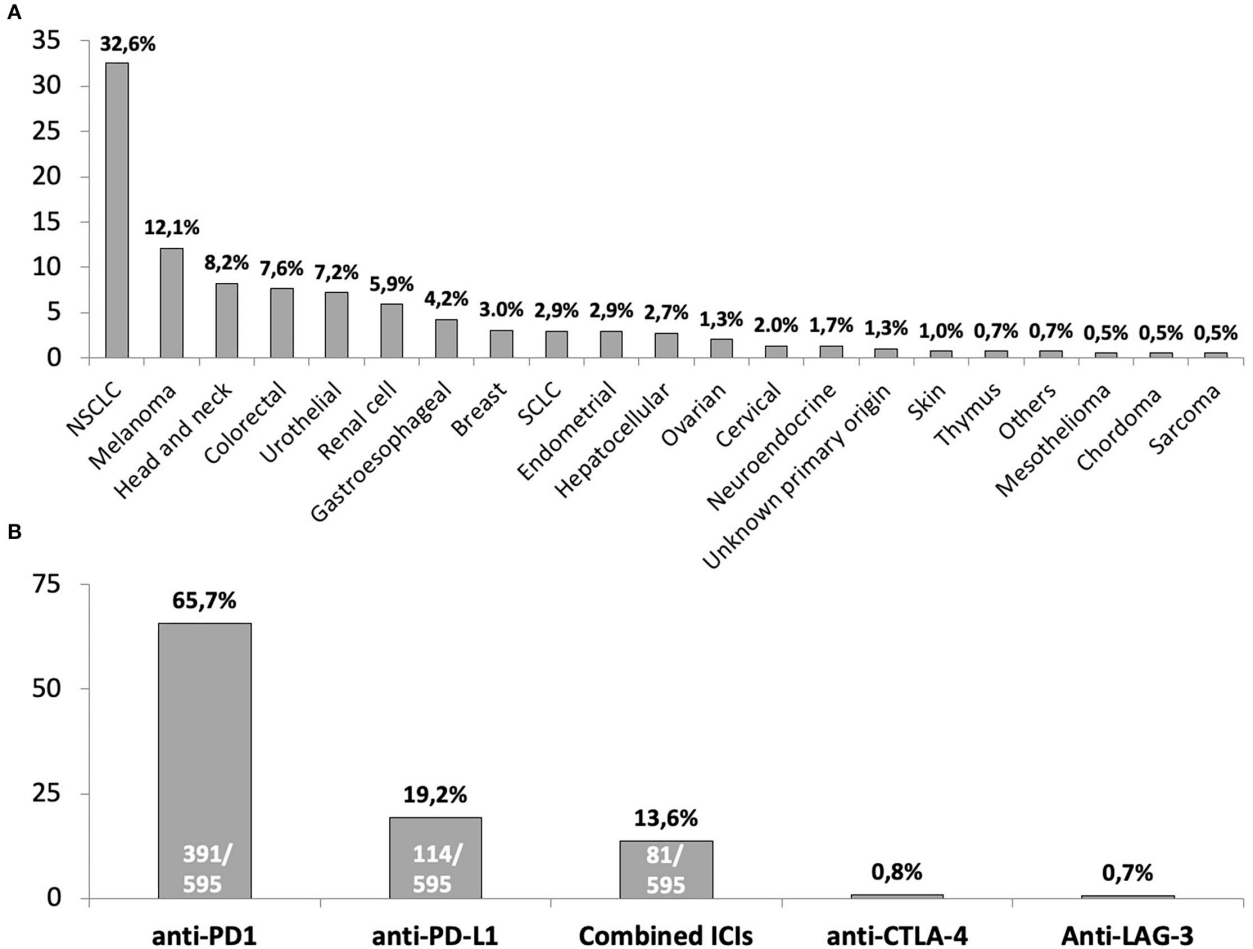
Summary of underlying cancers for immune checkpoint inhibitors therapy **(A)** and immune checkpoint inhibitors scheme prescribed to included patients **(B)**. NSCLC, non-small cell lung cancer; SCLC, small cell lung cancer. The four (0.7%) subjects included in the “*Others*” tumors category comprised: 1 paraganglioma, 1 solitary fibrous tumor, 1 thyroid, 1 Hodgkin Lymphoma. In the case of monotherapy with anti-CTLA-4 or anti-LAG-3, the absolute number of subjects who received these schemes of ICIs were 5 and 4, respectively.

During the 2-year period of study, 595 individuals received ICIs. The majority were male (61.2%), with a median age of 64 years. Forty-three (7.2%) subjects had a history of liver disease, mainly alcohol, and metabolically associated fatty liver disease, though only 15 (2.5%) presented signs of liver cirrhosis. The main characteristics of patients are summarized in Table 1. The most prevalent tumors were lung cancer, melanoma, head and neck, and colorectal which account for up to 60% of cancers, as shown in Figure 1A. Approximately half of the cohort (53.2%) had been previously treated with chemotherapy, and up to 15% had already received a prior line of therapy including ICIs.

**Table 1:** Main characteristics of patients treated with immune checkpoint inhibitors (*N* = 595).

**Patients' characteristics**	
**Male gender**	364 (61.2%)
**Age (years)**	64 (57-72)
**Race**	
Caucasian	582 (97.8%)
African/Hispanic/Asian	4 (0.7%)/8 (1.3%)/1 (0.2%)
**Underlying liver disease**	43 (7.2%)
Underlying liver cirrhosis	15 (2.5%)
**Liver metastasis**	123 (20.7%)
**Previous oncological therapy**	
Previous chemotherapy	316 (53.2%)
Previous immunotherapy	90 (15.2%)
**Current immunotherapy**	
Combined systemic therapy (all)	194 (32.6%)
Chemotherapy	104 (17.5%)
Tyrosine kinase inhibitor	21 (3.5%)
IL-2 agonist	14 (2.4%)
VEGF inhibitors	14 (2.4%)
Inducible Co-Stimulator (ICOS)	12 (2.0%)
MET inhibitors	8 (1.3%)
PARP inhibitors	7(1.2%)
**Corticoids at the beginning of ICIs**	11 (1.8%)

*Factors are expressed as n (%) or median (IQR)*.

The current scheme of immunotherapy is summarized in Figure 1B. The vast majority (84.9%) of individuals received monotherapy with either an anti-PD1 or anti-PD-L1 and 13.6% a combination of anti-PD-1 and anti-CTLA-4 agents. Concomitant systemic therapy was given to 194 (32.6%) subjects, with chemotherapy the most common (104, 17.5%), followed by tyrosine-kinase inhibitors (21, 3.5%) (Complete list in Supplementary Table 1). Twenty-three (3.9%) subjects had received corticoids in the past. Overall, 73 (12.3%) patients from the cohort received corticoids, either at the beginning (11, 1.8%) or during the course of immunotherapy. Median duration of immunotherapy at the time of the study was 9.4 months (range 1–86.3).

### Viral Hepatitis Screening Prior to Immunotherapy

**Figure 2:**
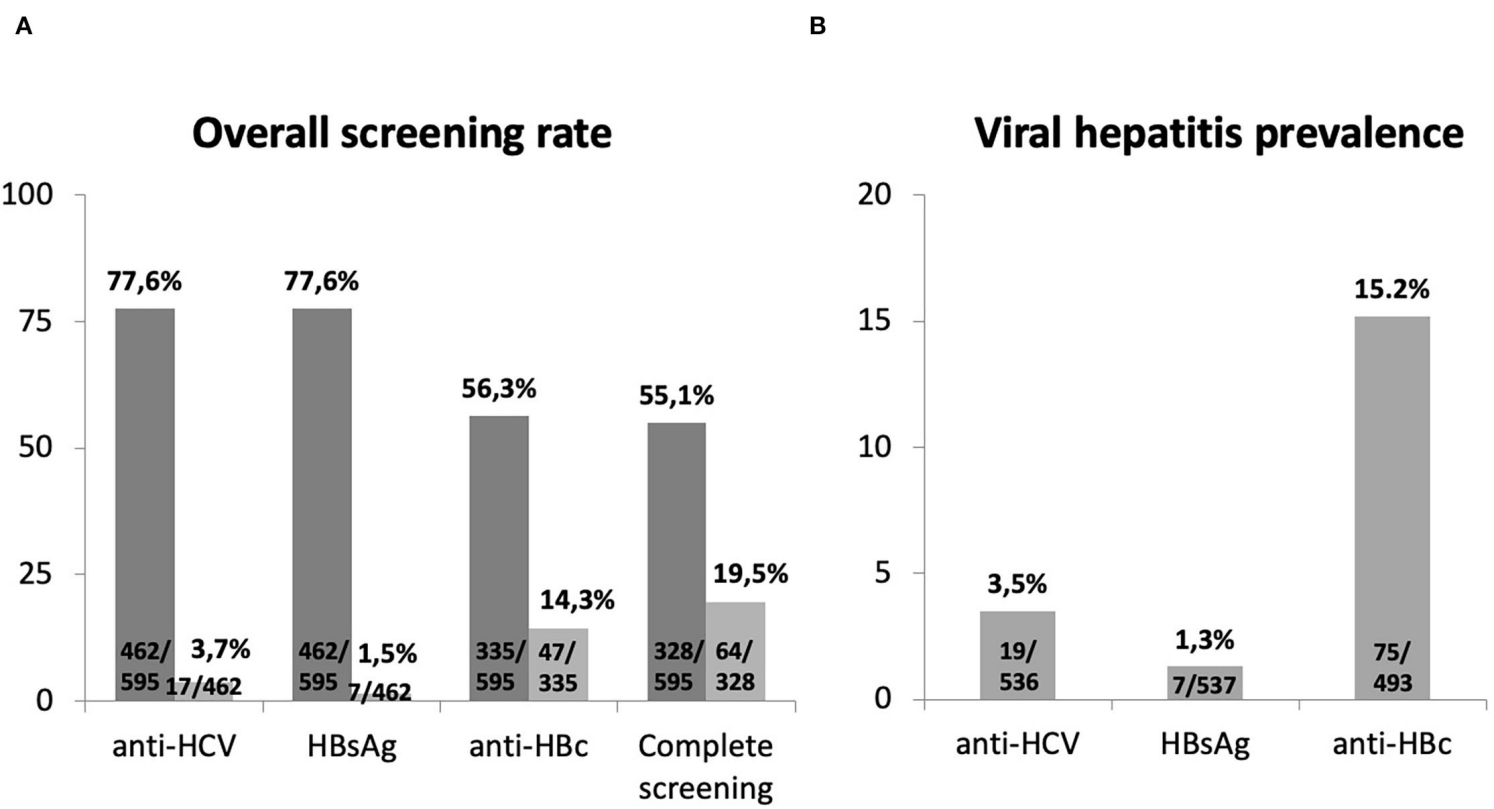
**(A)** Rate of viral hepatitis markers ordered prior to the beginning of ICIs (dark gray) and the results from these tests (light gray). **(B)** Overall viral hepatitis prevalence after the prospective request of viral hepatitis markers in alive patients on ICIs. *Complete viral hepatitis testing* refers to the combination of HBsAg, anti-HBc, and anti-HCV.

The percentage of patients with viral hepatitis screening previous to ICIs is summarized in Figure 2A. Overall, 328 (55.1%), subjects had *complete viral hepatitis testing* prior to ICIs, a percentage lower than the 392 (65.9%) subjects screened for HIV (*p* < 0.001). The percentage of subjects with ordered *complete viral hepatitis testing* prior to ICIs was higher among those with HIV results (67.1 vs. 32.0%, *p* < 0.001). Likewise, data on *complete viral hepatitis* was more frequent in patients who had been previously treated with either chemotherapy (59.6 vs. 50.0%, *p* = 0.012) or immunotherapy (72.2 vs. 52.1%, *p* < 0.001).

**Figure 3:**
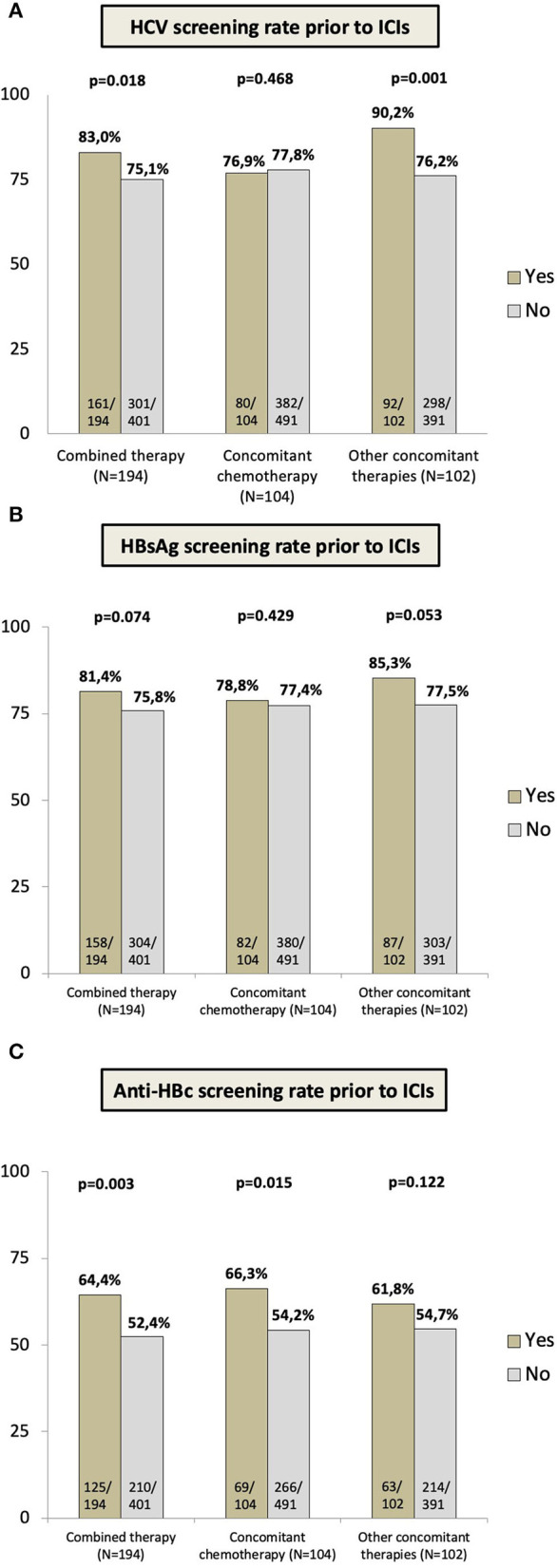
Rate of viral hepatitis testing ordered previous to ICIs starts according to the prescription of concomitant medication (All combined therapy; concomitant chemotherapy; concomitant systemic therapy different from chemotherapy mainly tyrosine-kinase inhibitors, IL-2 agonist, inducible co-stimulators-ICOS and anti-VEGF drugs as summarized in Supplementary Table 1: **(A)** hepatitis C virus screening prior to ICIs. **(B)** HBsAg screening prior to ICIs. **(C)** anti-HBc screening prior to ICIs.

Viral hepatitis screening differed in relation to the type of concomitant medications. The anti-HCV request was higher in subjects undergoing combined therapy (*p* = 0.018), mainly due to the greater awareness among those of systemic therapies different from chemotherapy (90.2 vs. 76.2%, *p* = 0.001) as shown in Figure 3A. These findings were also observed in the case of HBsAg, with a tendency to higher screening rates in individuals on *other* concomitant therapies (85.3 vs. 77.5%, *p* = 0.053) (Figure 3B). However, in the case of anti-HBc, the rate of screening was higher among individuals receiving concomitant chemotherapy (*p* = 0.015) and globally among those on combined systemic therapy (*p* = 0.003) as shown in Figure 3C. No differences were observed in the rate of viral hepatitis screening in the cohort of 26 individuals with current or previous therapy with corticoids (anti-HCV: *p* = 0.543, HBsAg: *p* = 0.543, anti-HBc: *p* = 0.227, *complete viral hepatitis testing: p* = 0.192).

### Prevalence of Viral Hepatitis Among Subjects Undergoing Immunotherapy

The prospective screening of those individuals without previous data and still alive at the time of the study revealed an overall prevalence of hepatitis markers of 3.5% for anti-HCV, 1.3% for HBsAg, and 15.2% for isolated anti-HBc (Figure 2B). This prospective search allowed the identification of two unknown cases of HCV infection and 28 isolated anti-HBc-positive subjects. No additional cases of HBsAg were detected.

Concerning hepatitis C, viral load was requested by the treating physician in 16 (84.2%) cases. In the remaining anti-HCV cases it was performed later and linked to the present study. All except 2 (11.5%) out of the 17 anti-HCV positive subjects had undetectable HCV-RNA, largely due to previous antiviral therapy. The two individuals with detectable HCV-RNA presented hepatocellular carcinoma. Among the seven HBsAg-positive patients, HBV DNA was tested in 6 (85.7%) prior to the start of immunotherapy and it was detectable in five cases. Among the 75 isolated anti-HBc-positive individuals HBV-DNA was requested in only 26 (34.7%) and just one patient presented a detectable viral load (28 IU/mL).

### Viral Hepatitis Reactivation

No cases of HCV reactivation were detected. Antiviral therapy were not initiated in the two patients with detectable HCV-RNA during immunotherapy and no changes in HCV-RNA levels were observed. Likewise, HCV-RNA remained undetectable in all subjects with prior resolved hepatitis C during a median 8-month ICIs therapy (range, 2–35).

HBV reactivation was not observed in any of the 7 HBsAg-positive cases. Five (71.4%) received antiviral prophylaxis during ICIs therapy. Four of these five subjects presented detectable HBV-DNA at baseline (median value of 104 IU/mL; range 0–11,475 IU/mL). In all patients on antiviral prophylaxis, HBV-DNA became and remained undetectable during ICIs therapy. In the 2 subjects who did not receive antiviral prophylaxis, baseline HBV-DNA values were 12.000 and 11.300 IU/mL, respectively. One of these patients died 2.7 months after the beginning of immunotherapy due to cancer progression and no HBV-DNA determination was available within this time. The other case completed 17 cycles of an anti-PD1 agent, but no viral load was determined during the course of ICIs despite the fact that the patient presented increased ALT levels throughout immunotherapy. HBV-DNA after ICIs discontinuation was 2,450 IU/mL.

HBV reactivation was not detected in 61 out of 75 anti-HBc-positive individuals who had periodical determinations of HBsAg and HBV-DNA during a median ICI therapy of 11 months. None of them received antiviral prophylaxis except for an HIV co-infected patient who underwent a Tenofovir-containing antiretroviral regimen. The only patient with a baseline detectable HBV-DNA remained similar throughout immunotherapy despite the lack of antiviral prophylaxis (last HBV-DNA value 20 IU/mL).

## Discussion

Herein we present novel results on the rate of screening of Oncologists for the risk of viral hepatitis associated with immunotherapy, with just 55.1% of patients having complete screening prior to ICIs. Moreover, we provide prospective data on the risk of HBV reactivation in a cohort of 61 subjects with past HBV infection (isolated anti-HBc+), with no cases meeting the criteria for reactivation during a median follow-up of 11 months of ICIs. To our knowledge, this is the first work focusing on the rate of viral hepatitis tests performed prior to immunotherapy, a useful tool to assess the awareness of ICIs' prescribers of the potential risk of immunotherapy on patients with chronic viral liver diseases.

This issue has been widely explored and discussed in the setting of chemotherapy, especially in those schemes including rituximab, an anti-CD20 agent. The high rates of HBV reactivation in patients on rituximab-containing chemotherapies led to the Food and Drug Administration and European Medicines Agency recommendations of HBsAg and anti-HBc screening prior to immunosuppression, to identify individuals with criteria for antiviral prophylaxis to avoid reactivation. As reported by the Anderson Center (USA), these guidelines had a huge impact in the hematology setting, with a rate of screening of over 70% among individuals with hematologic malignancies (15). However, the percentage of tests among patients with solid-organ tumors remained very low (10%) despite these recommendations (15). Data from our area produced similar results, with 60.5% of hematological patients with complete viral hepatitis tests ordered prior to chemotherapy, a percentage that rose to roughly 88% when an electronic-alert system was set up (16).

In comparison to standard chemotherapy, data on the risk of HBV reactivation in the setting of oncological immunotherapy is scarce, since all patients in the registry study of the ICIs were on NAs. Furthermore, given the mechanism of action of the ICIs, these drugs may play a role in the treatment of chronic hepatitis B. For instance, the efficacy of nivolumab, an anti-PD1 agent, has been tested in HBV virological-suppressed patients, though the reported impact on HBsAg levels after a single dose was modest (17). The exponential use of immunotherapy in clinical practice led to the emergence of isolated clinical cases reporting HBV reactivations in patients undergoing ICIs (8, 18). Retrospective data from 114-HBsAg+ individuals from China revealed a 17.2% rate of HBV reactivation among those treated with anti-PD1 agents in case of the absence of concomitant antiviral prophylaxis (7). More recently, a retrospective cohort including 511 HBsAg-positive subjects revealed HBV reactivation rates of 0.4 and 6.4% in those with and without antiviral prophylaxis, respectively, emphasizing the importance of HBV prophylaxis (19).

To date, experience on the possible impact of ICIs on individuals with resolved HBV infection has scarcely been explored. In this regard, Shah and Kothapalli reported no changes in HBV viral load among eight and five anti-HBc+ subjects treated with ICIs without antiviral prophylaxis (20, 21). These preliminary results are in line with ours, where no cases of HBV reactivation were observed among the 61 anti-HBc+ individuals with prospective data on HBV markers. For the time being, the current evidence on the extremely low risk of HBV reactivation among subjects with resolved HBV infection recommends against the use of antiviral prophylaxis in the case of therapy with ICIs.

Regarding HCV, despite the high percentage of cure achieved by the direct-acting antivirals (DAA) therapy and the efforts of micro and macro elimination programs for diagnosis and linkage to care of HCV-infected individuals, published data on screening among solid-organ cancer subjects on chemotherapy revealed a rate as low as 14% (22). In our cohort, 3.5% of patients were anti-HCV positive, a prevalence higher than that reported among the general population in our setting (23), probably due to the inclusion of individuals with high-risk factors for HCV exposure, such as those with hepatocellular carcinoma, head and neck, and lung cancer. As reported with HBV, literature on ICIs and chronic hepatitis C is also limited. In our cohort, just two patients presented active HCV infection, with the rest showing undetectable viral load, the majority after the achievement of sustained virological response through DAAs. No changes in HCV-RNA were observed in the two patients with detectable viremia at the beginning of ICIs, neither was there a relapse of HCV in patients with baseline undetectable HCV-RNA. This observation is in line with preliminary results from both anti-PD1 and anti-CTLA-4 agents, where even some transient reductions in HCV RNA were reported (11, 24). More recently, in a matched cohort study of nivolumab, an anti-PD1 agent, for renal cell carcinoma, no impact on HCV-RNA was described in 14 individuals with HCV infection (25).

Our study has some limitations. This is a retrospective and single-hospital-based study and therefore data on some patients were missed. However, we report novel and interesting results on the degree of awareness of Oncologists about the risk of prescribing ICIs for patients with viral hepatitis according to the rate of pre-treatment ordered screening. Furthermore, the prospective gathering of tests in patients with a lack of results resulted in data on the prevalence of the viral hepatitis marker among patients undergoing oncological immunotherapy and the risk of HBV reactivation in this population.

In summary, herein we report novel results on the screening of viral hepatitis among immunotherapy prescribers, revealing that HBV and HCV screening prior to ICIs start is suboptimal. Though the rate of viral hepatitis reactivation in this population seems extremely low, efforts should be made to optimize viral hepatitis screening prior to immunotherapy for the selection of candidates for either antiviral prophylaxis or periodical check-up.

## Data Availability Statement

The raw data supporting the conclusions of this article will be made available by the authors, without undue reservation.

## Ethics Statement

The studies involving human participants were reviewed and approved by Vall d'Hebron Hospital Ethics Committee. Written informed consent for participation was not required for this study in accordance with the national legislation and the institutional requirements.

## Author Contributions

LA, JB, and LR-O contributed equally to this work by collecting the data and helping write the manuscript. MR-B guided the data collecting, performed the statistical analysis, and wrote the manuscript. AB-D, AC-P, EM-C, CO-V, ND-M, MC, and AF helped to discuss the results. MB supervised the final manuscript. All authors contributed to the article and approved the submitted version.

## Conflict of Interest

The authors declare that the research was conducted in the absence of any commercial or financial relationships that could be construed as a potential conflict of interest.

## Publisher's Note

All claims expressed in this article are solely those of the authors and do not necessarily represent those of their affiliated organizations, or those of the publisher, the editors and the reviewers. Any product that may be evaluated in this article, or claim that may be made by its manufacturer, is not guaranteed or endorsed by the publisher.
