# Supplementary material for: The Low Incidence of Viral Hepatitis Reactivation Among Subjects on Immunotherapy Reduces the Impact of Suboptimal Screening Rate
Source: Front Med (Lausanne). 2022 Jul 15;9:916213. doi: 10.3389/fmed.2022.916213 (PMC9335294; doi:10.3389/fmed.2022.916213)
Supplement: Supplementary file 1 [file Table_1.DOC]

**Supplementary table 1**. Summary of concomitant systemic therapy different from chemotherapy (N=102)

| **Family drug** | **N(%)** |
| --- | --- |
| Tyrosine-kinase inhibitors | 21 (20.6) |
| IL-2 agonist | 14 (2.4) |
| Inducible co-stimulators (ICOS) | 14 (2.4) |
| Antagonists of Vascular Endothelial Growth Factor (Anti-VEGF) | 12 (2.0) |
| Mesenchymal-epithelial transition factor inhibitors (MET inhibitors) | 8 (1.3) |
| Peroxisome proliferator-activated receptors inhibitors (PPAR inhibitors) | 7 (1.2) |
| Interleukin inhibitors | 4 (0.7) |
| Lysine specific demethylase 1 (LSD1) inhibitor | 4 (0.7) |
| Anaplastic lymphoma kinase (ALK) inhibitors | 3 (0.5) |
| MEK inhibitor | 3 (0.5) |
| Arginase inhibitors | 3 (0.5) |
| Talimogene laherperepvec (T-VEC) | 2 (0.3) |
| T cell immunoglobulin and ITIM domain (TIGIT) inhibitor | 2 (0.3) |
| Kirsten rat sarcoma (K-ras) Inhibitor | 1 (0.2) |
| Inhibitors of apoptosis (IAPs) | 1 (0.2) |
| Adenosine signaling through A2A receptors (A2AR) inhibitors | 1 (0.2) |
| Cyclin-dependent kinases (CDKs) inhibitors | 1 (0.2) |
| Toll-like receptor 4 (TLR-4) inhibitors | 1 (0.2) |
